# MPrime: efficient large scale multiple primer and oligonucleotide design for customized gene microarrays

**DOI:** 10.1186/1471-2105-6-175

**Published:** 2005-07-13

**Authors:** Eric C Rouchka, Abdelnaby Khalyfa, Nigel GF Cooper

**Affiliations:** 1 Department of Computer Engineering and Computer Science, Speed School of Engineering, University of Louisville, Louisville, Kentucky, USA; 2 Department of Anatomical Sciences and Neurobiology, University of Louisville School of Medicine, Louisville, Kentucky, USA; 3 Bioinformatics Research Group, University of Louisville, Louisville, Kentucky, USA

## Abstract

**Background:**

Enhancements in sequencing technology have recently yielded assemblies of large genomes including rat, mouse, human, fruit fly, and zebrafish. The availability of large-scale genomic and genic sequence data coupled with advances in microarray technology have made it possible to study the expression of large numbers of sequence products under several different conditions in days where traditional molecular biology techniques might have taken months, or even years. Therefore, to efficiently study a number of gene products associated with a disease, pathway, or other biological process, it is necessary to be able to design primer pairs or oligonucleotides en masse rather than using a time consuming and laborious gene-by-gene method.

**Results:**

We have developed an integrated system, MPrime, in order to efficiently calculate primer pairs or specific oligonucleotides for multiple genic regions based on a keyword, gene name, accession number, or sequence fasta format within the rat, mouse, human, fruit fly, and zebrafish genomes. A set of products created for mouse housekeeping genes from MPrime-designed primer pairs has been validated using both PCR-amplification and DNA sequencing.

**Conclusion:**

These results indicate MPrime accurately incorporates standard PCR primer design characteristics to produce high scoring primer pairs for genes of interest. In addition, sequence similarity for a set of oligonucleotides constructed for the same set of genes indicates high specificity in oligo design.

## Background

Recent advances in DNA sequencing technologies have resulted in the availability of the assembly of a number of genomes at various stages. As of December 24, 2004, the Genomes OnLine Database [1] lists 1,245 on-going or completed genome projects. Included are the genomes of human [2,3], mouse [4], Norwegian brown rat [5], fruit fly [6], fugu [7], chimpanzee, and zebrafish [8]. In addition, various projects have been concerned with the curation of the genic regions of these genomes, most notably RefSeq [9].

Advances in microarray technology [10] have made it possible to simultaneously study the expression levels of thousands of genes under a given condition. Since a molecular biologist may be interested in studying a particular subset of genes in a given genome using microarray experimentation, it becomes necessary for the scientist to design complementary sequences to uniquely identify the genes of interest for either a cDNA product [10] or synthetic oligonucleotide [11-13] approach.

Custom microarray experimentation involves several steps, each of which must be performed in a timely manner in order to avoid becoming a bottleneck in the system. The steps involved are: determination of target genes; designing primers or oligonucleotides to create products uniquely identifying the target genes; spotting of the products on the microarray slide; detection of the presence of the gene under a given condition; and data analysis. MPrime focuses on the primer and oligonucleotide design stage in the microarray experimentation process.

### Comparison to existing primer and oligo design software

Within the past few years, several algorithms for primer design have become available [14-21] including some specifically targeting whole genome analysis for microarrays [22-24]. In addition, more recent publications have become available discussing methods for oligo design [25-30], due to the emergence of custom oligonucleotide arrays. However, many of the primer design programs do not consider product similarity for cross hybridization, in part due to the lack of available genomic sequence at the time of their publication (see table 1).

**Table 1:** Comparison of properties of MPrime with various primer and oligonucleotide design programs.

**Program**	**Reference**	**Primers**	**Oligos**	**Large Scale Design**	**Sequence Similarity Avoidance**
Primer Master	Proutski and Holmes (1996) [19]	**X**			
PRIMO	Li, et al. (1997) [17]	**X**			
GenomePRIDE	Haas, et al. (1998) [14]	**X**	**X**	**X**	**X**
Primer3	Rozen and Skaletsky (2000) [21]	**X**		**X**	
DOPRIMER	Kampke et al., (2001) [15]	**X**		**X**	
PrimeArray	Raddatz et al. (2001) [20]	**X**		**X**	
GST-PRIME	Varotto et al. (2001) [23]	**X**		**X**	
ProbeSelect	Li and Stormo (2001) [25]		**X**	**X**	**X**
PROBE	Pozhitkov and Tautz (2002) [27]		**X**	**X**	**X**
PRIMEGENS	Xu et al. (2002) [24]	**X**		**X**	**X**
OligoWiz	Nielsen et al. (2003) [26]		**X**	**X**	**X**
OligoArray 2.0	Rouillard et al. (2003) [29]		**X**	**X**	**X**
OligoPicker	Wang and Seed (2003) [30]		**X**	**X**	**X**
ROSO	Reymond, et al. (2004) [28]		**X**	**X**	**X**
MPrime	2005	**X**	**X**	**X**	**X**

There are a number of limitations to these approaches. Some of the algorithms find a primer or oligo from a single sequence at a time. Such an approach is time consuming and prone to human error, since the results must be transferred one at a time. Other algorithms may consider multiple sequences, yet fail to consider a sequence similarity score, resulting in products which do not uniquely identify genes. Some approaches pre-compute a set of products for a given genome, which works well if the complete genome is available, but behaves poorly in an adaptive environment. Very few of these approaches allow the user to specify whether they would like to design either primers or oligos, limiting them to one or the other.

MPrime attempts to overcome each of these limitations by allowing for efficient large scale multiple product design. MPrime approaches primer and oligo design by looking at a user-defined subset of genes of interest identified by either a GenBank [31] or RefSeq [9] accession number, by a gene name, or by the raw fasta sequence data. When the user specifies a particular genome of interest, MPrime will search through that genome to ensure that the products chosen will uniquely identify the gene the user is trying to identify.

Due to the methods employed, MPrime can be useful in large-scale primer and oligo design. Two software packages, GenomePRIDE [14] and GST-PRIME [23], have been described as offering large-scale, genome-wide design of oligomers and primer pairs for the construction of custom microarray products. MPrime provides advantages to each of these approaches.

GST-PRIME allows for an automated, genome-wide design of primer pairs in a similar fashion to MPrime. MPrime improves upon the functionality of GST-PRIME by allowing for the construction of gene-specific oligos for use with oligo-based custom microarrays. In addition, while GST-PRIME allows for the detection of primer pairs for a cDNA based chip on a whole genome, users of the system are limited to only choosing genes associated with a protein GenBank Identifier (GI) number. This inherently imposes limitations for the user. For instance, computationally predicted genes, genes supported by biological evidence (ESTs for example), or pre-published gene sequences cannot be used. MPrime avoids these complications by allowing the user to obtain the gene information directly from the GI number, or optionally from a raw fasta formatted sequence.

GenomePRIDE is the only known software package that incorporates high throughput primer pair and oligo design. MPrime distinguishes itself from GenomePRIDE by taking into account several factors, including GC content, secondary structure loop formation, and the presence of a GC clamp. While omitting these calculations makes the detection of primers and oligos much more efficient, it can also potentially lead to problems with both PCR product amplification and probe to product binding affinities. In addition, MPrime is freely available via a web interface, whereas GenomePRIDE has an associated cost of 150 euros for academic institutions.

## Implementation

### Selection of genic regions

The first step in primer design is to decide the regions to study. The interests of our collaborating lab lie in collating sets of mammalian programmed cell death genes related to neurological diseases such as Alzheimer's, Parkinson's, and Huntington's for use in microarray analyses. Therefore, an interface has been incorporated into MPrime in order to allow the user to retrieve genic regions by either a GenBank accession, gene name, or by a keyword. Retrieval by a keyword allows the user to have a general idea of the region of interest without having to spend time searching for an exact gene name or GenBank accession. Potential candidates are presented to the user. MPrime allows the user to refine their results by deciding on which candidate regions will be included before proceeding to primer or oligo design.

### Primer design calculations

Although there are potentially a number of applications for the use of MPrime, it was originally designed with the intent for the construction of primers for custom cDNA microarray chips. In order to obtain the primer products with a high throughput, multiplex PCR can be implemented as the underlying biological experimentation. To ensure that the multiplex PCR experiments function properly and the microarray experimentation produces results that can be meaningfully analyzed the primer products should adhere to the properties of uniformity, sensitivity, and specificity.

#### Product uniformity

Multiplex PCR [32] is a technique allowing for the amplification of two or more targets in an organism of interest by incorporating multiple sets of PCR primers into a single reaction. Since each set of primers is subject to the exact same reaction conditions, they must be defined uniformly in order to react to the thermo cycling in a similar fashion. The properties which need to be relatively uniform include primer melting temperature, primer G+C concentration, and primer length.

MPrime requires the user to input a range of primer melting temperatures along with an optimal melting temperature. Any primers designed are required to fall inside of this range. There are a number of different approaches to calculating the melting temperature (*T*_*m*_) of an oligonucleotide sequence, including an arbitrary method based on the formula *T*_*m*_ = 2 * (A + T) + 4 * (G + C) [33]; a long probe method based on the length and GC concentration [34]; and a nearest neighbor method based on dinucleotide interactions [35,36]. MPrime incorporates the nearest neighbor approach using the formula of Rychlik, et al. [36] where the *T*_*m *_is calculated as follows:



Where *ΔH *is the enthalpy for helix formation, *ΔS *is the entropy for helix formation, *c *is the molar concentration of the primer (set at 250 pM); *M *is the molar concentration of Na+ (set at 50 mM) and *R *is the gas constant (1.987 cal/degree * mol).

Calculation of the primer G+C concentration is a straight-forward calculation of the percentage of the primer oligo containing G's or C's. MPrime requires the user to input the minimum, maximum, and optimal G+C concentration for the primers. In addition, the user can specify a GC clamp length, or the number of G's or C's required at the 3' end of the primer. The GC clamp helps to promote primer end stability, resulting in more efficient and specific bonding of the primer to the amplicon template.

In addition, MPrime requires the user to enter information concerning the primer length. The minimum, maximum, and optimal primer lengths are required. The primer length uniformity along with the uniform G+C content helps to maintain a uniform primer melting temperature.

#### Product sensitivity

**Figure 1:**
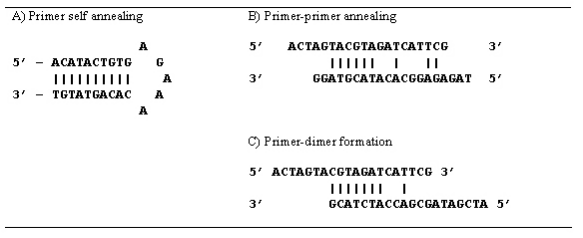
**Primer secondary structure formation. **Shown in figure 1A) is the secondary structure stem loop formation for the primer ACATACTGTGAGAAACACAGTATGT. Figure 1B) illustrates an example double-stranded structure formation between the primers ACTAGTACGTAGATCATTCG and GGATGCATACACGGAGAGAT. Note the run of six straight matches between the two sequences. Shown in figure 1C) is the primer-dimer formation between the primers ACTAGTACGTAGATCATTCG and GCATCTACCAGCGATAGCTA. Note the 3' end of the second primer has a run of seven straight matches to the first. The scores for each of these is based on the formula 4 * |G + C| + 2 * |A+T|. For figure 1A) there are 6 A-T base pairs, and 4 G-C base pairs, yielding a score of 28. For figure 1B), there are 6 A-T base pairs, and 3 G-C base pairs, yielding a score of 24. For figure 1C), there are 4 A-T base pairs and 3 G-C base pairs on the 3' end of the second sequence, yielding a score of 20.

PCR amplification can potentially fail due to either self or paired annealing. There are three main cases to be considered. In the first case, if either of the individual primers have a large number of consecutive complementary bases within their sequences they can potentially self-anneal to form a secondary structure such as a stem loop (see figure 1A). In the second case, complementary bases in paired primers can interact in their mid-regions to form partial double stranded structures (see figure 1B). The final case occurs when the primers interact with each other at the 3' end of either primer to produce primer-dimers (see figure 1C).

One problem that can occur in product design is that either the products or the corresponding probe sequences could form secondary or tertiary structures, thus causing them to fail to interact, resulting in an apparent low signal. In order to overcome these problems of unwanted annealing between primers and products, MPrime incorporates a scoring scheme for paired-end and self-end annealing to reduce primer dimers; paired annealing to reduce partial double stranded structures; and self annealing to reduce secondary structure formation of single stranded sequences within both products and individual primers. These scoring schemes are adapted from Kampke, et al. [15] where each G-C base pair is assigned a score of 4 and each A-T base pairing is assigned a score of 2.

#### Product specificity

It is important that each primer set results in only the product of interest, and that each product uniquely identifies the gene being studied. In order to build in these constraints, MPrime searches the primers against a RefSeq [9] database for the organism being studied using wublastn [37]. The highest scoring sequences should be the genic region of interest; all other similar, suboptimal alignments will result from spurious hits. In order to assure that the product designed is specific to the gene of interest, the blast scores for all database matches are considered, and a specificity score is computed as the percent identity of the match multiplied by the percent of the product covered by the match. The blast results are then resorted based on this specificity score. The native locus should have a score of 1 (100% identity * 100% coverage). The next highest scoring segment is then taken to give an idea of the specificity of the designed product. For instance, if the product is completely unique, the second highest score will be 0. If the next highest scoring segment has an 80% identity of 60% of the sequence, the score would be 0.48. The product specificity score is weighted and calculated into the overall primer score. Note that in some instances, a gene will have duplicate entries in RefSeq. This may result in the product specificity score of 1 for all possible products. In such a case, the best scoring primer set is reported along with the product specificity score. In these cases, it is up to the user to determine whether or not such a primer set should be used.

MPrime incorporates an overall scoring scheme for each pair of primers. The basis behind this scheme is to find the best scoring primer pair with the smallest deviation from the overall optimal values. The smaller the score is, the more likely it is that the primer pair will function as desired. Each of the parameters entered is weighted evenly, with the exception that a larger weight is associated to product specificity. Similar scoring schemes have been proposed in Kampke, et al. [15] and are used in Primer3 [21].

### Oligonucleotide calculations

MPrime also has the capability of calculating oligonucleotides for sequences of interest. The scoring scheme for determining the optimal oligonucleotides is very similar with the exception that there are no primer-primer interactions that can occur. With oligonucleotides, the similarity scores are weighted slightly higher, and are based on the actual length of the oligos desired. Typically, the 3' end of genes tends to be more variable and therefore more unique in their sequence. In addition, MPrime has the added feature of allowing the user to specify a region in which the oligo must be located.

### Sequence input into MPrime

Sequences can be input into MPrime in one of four ways: by gene name, by GenBank accession number, by keyword, or by the raw fasta sequence data. The user can specify whether they are interested in sequences from the human, rat, mouse, fruit fly, or zebrafish genome. MPrime will then search for the sequence of interest, and return sequence records from RefSeq and the appropriate GenBank databases matching the search string. If the GenBank accession is given, MPrime first checks to see if there is a gene matching exactly.

Once the regions of interest are decided upon, the sequences are retrieved from local copies of either RefSeq or GenBank. For cDNAs, these sequences are then searched in order to retrieve primer pairs adhering to a set of guidelines. The primer length, G+C content, melting temperature, self-hybridization characteristics, and primer-primer hybridization characteristics must be taken into account in order to allow the primers to effectively be used in PCR experiments [15]. In addition, information on the sequence product is taken in as well, including the product length, G+C content, and melting temperature. The MPrime software was developed using both Perl and C++.

Since the end goal is to create products for incorporation into gene microarray chips, it is extremely important to provide relatively uniform product lengths in order to provide equal concentrations of sequence products and to be able to distinguish between the expression levels of different genes within a single experiment. The MPrime interface allows the user to enter in a range of values along with an optimal product length.

After potential primer pairs are created, they are searched against the appropriate database of genomic sequence in order to ensure the primer pairs and subsequent products are unique. If the products on the microarray chip are to uniquely identify genes, then it is necessary to check that the resulting products do not represent domains repeated in several genes.

### Validation of MPrime

Sequence products produced by primer pairs selected from MPrime were validated using two independent techniques: (i) polymerase chain reaction and (ii) sequencing using the Beckman Coulter CEQ8000 Genetic Analysis System. Oligonucleotides constructed from MPrime were checked for their specificity by a nucleotide sequence search against *Mus musculus *sequences.

#### Polymerase chain reaction (PCR)

After primer pairs are determined, the sequence products are amplified using a 96 well plate DNA Engine Tetrad Thermal Cycler (MJ Research, Inc., Boston, MA). A MultiPROBE II Liquid Handler (Packard Instruments, Boston, MA) is used to help automate the process of creating the 96 well trays used in PCR amplification. The resulting products are then spotted on customized microarrays using a BioChip Arrayer (Packard Instruments).

PCR was performed using mouse brain total RNA (Clontech, Palo Alto, CA) and reverse transcribed into cDNA. RT-PCR was performed for a set of housekeeping genes and the primers used for RT-PCR were the same as those designed by MPrime program. Briefly, 5 μl of total RNA was used reverse transcribed using SuperScript II (Invitrogen, Carlsbad, CA) as described by the manufacture protocols. PCR reaction including 10 μl of cDNA template in a 100 μl reaction volume was amplified using DNA Engine Tetrad (MJ Research, Inc., Boston, MA). The thermal cycling profile consisted of 95°C for 3 min, 94°C for 30 s, 60°C for 30 s, 72°C for 30 s and 72°C for 7 min. Cycling kinetics were performed using 30 cycles. Amplified PCR reactions were separated on 2% agarose gel in the presence of ethidium bromide for visualization.

#### Sequencing

Purified mouse housekeeping genes products were sequenced using the CEQ 8000 Genetic Analysis System (Beckman Coulter Inc., Fullerton, CA). PCR was carried out using left primer and target cDNA from mouse brain as described above for 25 cycles. Sequences were then compared using a blast search against the corresponding GenBank and/or RefSeq entry for verification.

#### Oligonucleotide specificity

MPrime-computed oligonucleotides were compared against sequences from the organism *Mus musculus *using NCBI Blast [38]. The nucleotide-nucleotide Blast program blastn was used with default parameters. The advanced search option limiting to the *Mus musculus *dataset was used. Similar sequences reporting a bit score of 99.7 or higher (five mismatches in 70 bases) were reported as significant alignments for the purpose of oligonucleotide specificity.

## Results

**Figure 2:**
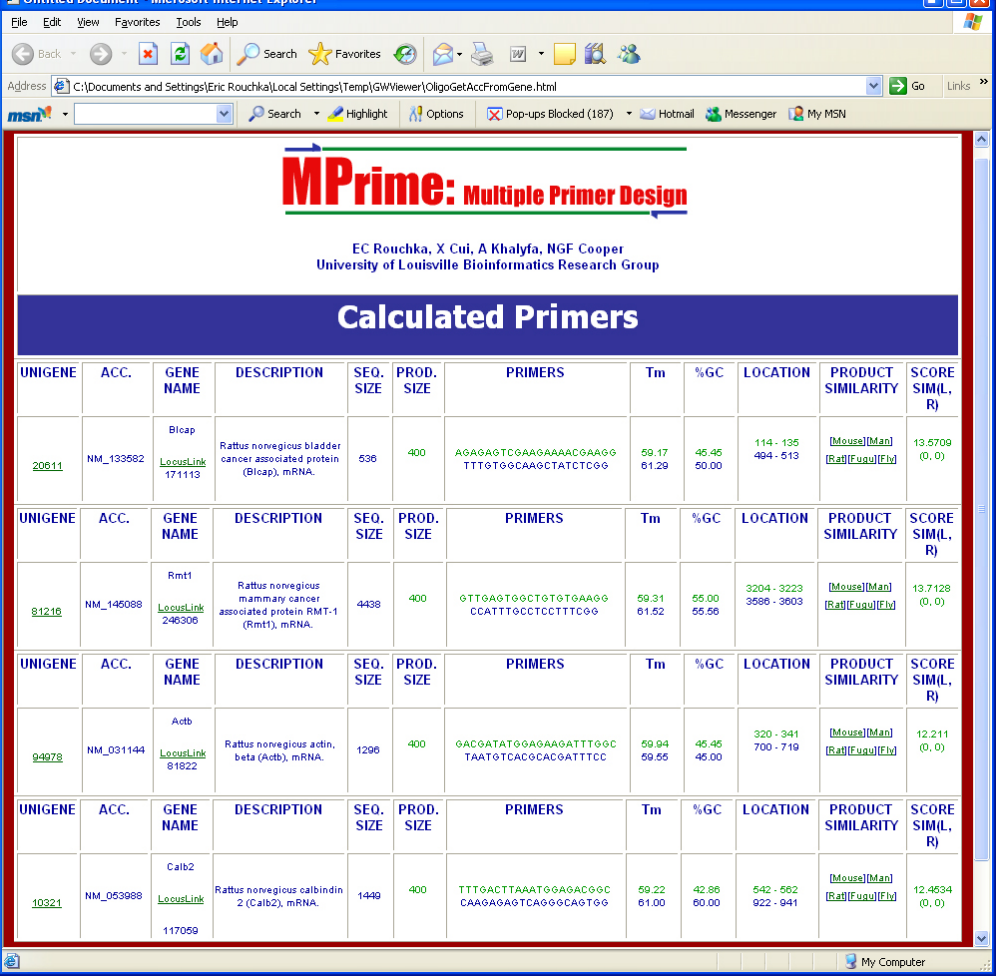
**Web interface and example of primer pair output. **The initial inputs were for the rat genome with GenBank accession NM_053986, gene ACTB, and keyword "cancer". The default values for the primer size, GC%, melting temperature, product size, and GC clamp length were used. The user was able to refine these choices to include the four genic regions indicated in the results. A similar screen is produced when oligonucleotides are chosen as well (not shown).

MPrime has allowed us to create multiple primers for related genic regions in a short span of time which are reported back to the user in the form of a web interface that can be stored as a tab delimited file that can be read into an Excel spreadsheet (figure 2). In our hands, detection of primers on a set of 138 rat actin, myosin and various muscle related genes with an average size of 2.7 KB finished in approximately 80 seconds on a dual processor AMD Athelon system with 1 GB of memory (results not shown). Analysis of the primers designed using the MPrime strategy compare well to the widely used Primer3 [21] software (results not shown).

**Figure 3:**
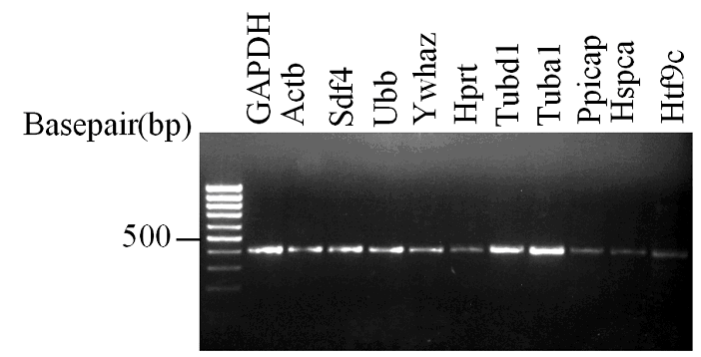
**PCR amplification results for eleven mouse housekeeping genes. **The resulting bands indicate single products with product sizes near 400 bases in length.

**Figure 4:**
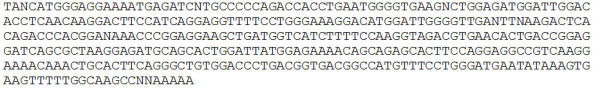
Sequencing results for the primer product for mouse stromal cell derived factor 4 (Sdf4) sequence.

**Figure 5:**
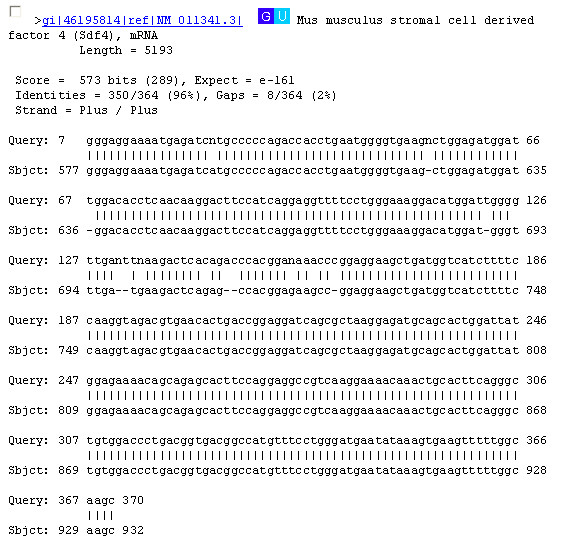
Comparison of the PCR product for the mouse stromal cell derived factor 4 (Sdf4) obtained from the MPrime PCR primers (Figure 4) to the RefSeq sequence NM_011341.3 [GenBank:46195814].

A set of eleven randomly chosen mouse housekeeping genes (GAPDH, Actb, Sdf4, Ubb, Ywhaz, Hprt, Tubd1, Tuba1, Ppicap, Hspca, and Htf9c) was chosen for study. The resulting MPrime primer pairs detected for each of these genes is located in Table 2. The results of the PCR amplification showing products on the order of 400 bases for each of the primer pairs for these genes are shown (Figure 3). Sequencing of these products and subsequent similarity searches led to the identification of uniquely identifiable regions for each of these eleven product pairs. Sample results for the stromal cell derived factor 4 (Sdf4) are shown in terms of both the sequence (Figure 4) and the resulting sequence similarity results (Figure 5). The remaining ten sequences show similar results in terms of both their sequence composition and sequence similarity, validating that the sample set of primers produces the desired products. Thus, MPrime provides an effective alternative to finding primers one gene product at a time.

**Table 2:** Sample set of genes and resulting primer pairs using MPrime1.0. The top sequence in each primer pair is the forward primer, the second sequence is the reverse primer.

**Gene Name**	**Product Size**	**Primer Pairs**
GAPDH	400	AATGTGTCCGTCGTGGATCT GGGTCTGGGATGGAAATTGT
Actb	400	GGCGCTTTTGACTCAGGATT AGTTGGGGGGACAAAAAAAA
Sdf4	400	AGACCTGCCAACCACTCATC TGCTTGCCAAAAACTTCACT
Ubb	400	TTCTGTGAGGGTGTGAGGGT TTTATCCTGGATCTTGGCCT
Ywhaz	400	ACAATGTTCTTGGCCCATGT AGGAAGAGGAGGAGGAAGGA
Hprt	400	TTATGCCGAGGATTTGGAAA AACCTTAACCATTTTGGGGC
Tubd1	400	TAAGATGCTGGGTGTCCGTA TAAGAGCTGGCTGTTGCTGA
Tuba1	400	GACCCTCGCCATGGTAAATA AATCCACACCAACCTCCTCA
Ppicap	400	ACTCCCTCCCTCTTTCCCTG CAGCAGAGAAAAGCTCCACC
Hspca	400	AGGAAACCCAGACCCAAGAC ACACCAAACTGGCCAATCAT
Htf9c	400	TGTGAATTCCTGGTCGGAGT TCTTTCTCTGTCCCTCCTCC

MPrime-calculated oligonucleotides of length 70 for the set of eleven housekeeping genes are shown (Table 3). Each oligo was designed to occur in the last 500 bases on the 3' end of the sequences. The oligos were searched against the *Mus musculus *sequences in NCBI's database using NCBI Blast [38] with the default parameters. Alignments resulting in five or fewer matches over the entire length of the oligo were reported and summarized (Table 4). The resulting sequence similarities and bit scores are given. A perfect bit score for an oligo of length 70 using the default blast parameters is 139. The results show that ten of the eleven housekeeping genes map uniquely to a single RefSeq gene sequence, with the exception being GAPDH. Nine of these ten also map to a single genomic locus, with the exception of Ubb, which maps to three separate loci (including the native locus). Ubb, Hprt, Tubd1, Tuba1, Hspca, and Htf9c also map to additional sequences not shown in the table. In each case, these additional sequences represented either redundant mRNA or cDNA sequences for the corresponding gene, or their corresponding chromosomal location. For these ten sequences, the results suggest MPrime produces highly specific oligonucleotide sequences.

**Table 3:** Computed 70-mer oligos for eleven mouse housekeeping genes using MPrime 1.3 default parameters.

**Gene name**	**70-mer oligonucleotide sequence**
GAPDH	ACTTTGTCAAGCTCATTTCCTGGTATGACAATGAATACGGCTACAGCAACAGGGTGGTGGACCTCATGGC
Actb	AATAGTCATTCCAAGTATCCATGAAATAAGTGGTTACAGGAAGTCCCTCACCCTCCCAAAAGCCACCCCC
Sdf4	TGTTAAAAGAAAACATGAAGAGAGCTGTGGCTCTAGCTCAGTGGTCGAACGCTGCCCAGCAAGTAAAACG
Ubb	CCTCCGTCTGAGGGGTGGCTATTAATTATTCGGTCTGCATTCCCAGTGGGCAGTGATGGCATTACTCTGC
Ywhaz	CTGTCACCGTCTCCCTTTAAAATCCTTCCTCCTCCTCTTCCTCCTCCTCCTCCTCCTCACATAATGATGG
Hprt	TTTTAGAAATGTCAGTTGCTGCGTCCCCAGACTTTTGATTTGCACTATGAGCCTATAGGCCAGCCTACCC
Tubd1	TTTGGACAGCTTTGCATTGTTGGAGCAAGTTGTTGCCAGTTATGGTAGTCTTGGACCCTAAGCCAAGAGG
Tuba1	GCTTCCACAGGGATGTTTATTGTGTTCCAACACAGAAAGTTGTGGTCTGATCAGTTAATTTCTATGTGGC
Ppicap	ATTGTATTCAAATGAAAATTTACTAGAAGGTTTCAGCCAGCACTCACTCCAGGACTGAGAGTCCCAGGGC
Hspca	TTAAAACAACCTGACAGGAATTCCCCAAGTGGCTTGTTTTCCAAAGTCCCGAGAACAACCCTAAGTTTCC
Htf9c	CAGACTCCGCACTGTGAGATGCTTATCCTGTTTGAGAGGATGCAACAACACCCCAATGGCATAGAAGCCC

**Table 4:** Blastn similarities to MPrime computed oligos. ^1^mRNA sequence from which the RefSeq sequence was derived.

**Gene name**	**GenBank identifier**	**Score (bits)**	**Notes**
GAPDH	**47607489**	139	RefSeq entry for GAPDH

Actb	***6671508***	139	RefSeq entry for Actb
	49865^1^	139	mRNA for beta actin
	26104752	139	beta actin cDNA
	191660	139	Mouse A-X beta actin mRNA
	22316184	99.6	Chromosome X genomic sequence
Sdf4	***46195814***	139	RefSeq entry for Sdf4
	47568754^1^	139	mRNA for Sdf4
	21953005	139	Chromosome 4 genomic sequence
	26097254	131	Unknown EST
Ubb	***6755918***	139	RefSeq entry for Ubb
	55177^1^	139	mRNA for Ubb
Ywhaz	***31981422***	139	RefSeq entry for Ywhaz
	29748001^1^	139	mRNA for Ywhaz
	1304165	139	mRNA for phosholipase A2
Hprt	***7305154***	139	RefSeq entry for Hprt
	193984^1^	139	mRNA for Hprt
	38348687	139	Chromosome X genomic sequence
Tubd1	***9790052***	139	RefSeq entry for Tubd1
	5813775^1^	139	mRNA for Tubd1
	17221230	139	Chromosome 11 genomic sequence
Tuba1	***6755900***	139	RefSeq entry for Tuba1
	202207^1^	139	mRNA for Tuba1
	202222	139	Tuba1 gene, 3' end
Ppicap	***6755143***	139	RefSeq entry for Lgals3bp (Ppicap pseudonym)
	297032^1^	139	mRNA for mama
	17017768	139	Chromosome 11 genomic sequence
	26348284	139	Ppicap cDNA
	397799	139	CyCAP mRNA
Hspca	***42476088***	139	RefSeq entry for Hspca
	12835986^1^	139	Hspca mRNA
Htf9c	***6680314***	139	RefSeq entry for Htf9c
	318976^1^	139	Htf9c mRNA
	3818382	139	Chromosome 16 genomic sequence

For GAPDH, there were not any oligonucleotides found in the 500 bases on the 3' end of the RefSeq sequence of length 70 that were unique to GAPDH. The highest scoring oligo resulted in 37 matches with a perfect bit score of 139. This is reflected in the high sequence similarity score reported by MPrime. For these 37 matches, fourteen were to predicted similar mRNAs; five were similar to GAPDH; and four were GAPDH mRNA. The remaining fourteen sequences were genomic matches to chromosome 2 (2 sequences) chromosome 3 (3), chromosome 4 (2), chromosome 7 (2), chromosome 8 (3), chromosome 10 (1), and chromosome 15 (1).

MPrime uses RefSeq as the default database for searching for gene sequences. In order to understand more fully what happens with GAPDH, RefSeq was examined more closely. A search of RefSeq with the constraint "Mus musculus similar to glyceraldehyde-3" "Mus musculus" [ORGN] results in 118 different sequences, many of which completely cover the GAPDH sequence used by MPrime. Since the GAPDH sequence is not unique within the set of RefSeq sequences, a unique 70-mer could not be detected.

## Discussion

In addition to the detection of primer products and single, long oligomers (on the order of 40–70 bases) that uniquely identify a gene of interest, individual researchers may want to design multiple probes for a single gene. Such an approach can help to limit the effects of cross-hybridization, the presence of multiple gene isoforms, and single nucleotide polymorphisms. An approach to design multiple short oligonucleotide probes (on the order of 25 bases long) has previously been presented [39]. MPrime presents the user the option of reporting back more than one resulting primer product or oligonucleotide for each gene. These results are reported in order based on their optimal score, with a score of 0 having the least deviation from the optimal user-selected conditions.

Researchers may additionally be interested in looking at multiple overlapping probes for each gene to produce tiled arrays that can help to detect the presence of different isoforms or single nucleotide polymorphisms present in an organism [40]. This option is not currently available in MPrime, but is a planned addition in a future release.

## Conclusion

MPrime has proven to be an efficient and effective tool for both primer and oligonucleotide design. The methodology behind MPrime allows molecular biologists to construct a large number of primers and/or oligonucleotides for genes that need to have consistent properties. In addition, multiple sub-optimal results can be reported and tested. Preliminary tests on a set of housekeeping genes indicate that primer products obtained by MPrime-designed primer pairs produce uniquely identifying regions. Oligonucleotides designed by MPrime also appear to produce highly specific segments as indicated by similarity searches to a number of databases. As more organisms become sequenced and customized gene microarrays become cheaper, the availability of interactive tools such as MPrime to design sequences for custom use will become even more important.

## Availability and requirements

Project name: MPrime: multiple primer design

Project home page: 

Operating system: Platform independent (developed using Perl and C++)

Other requirements: MPrime is a freely available to both academic and commercial users as a web-based form.

## List of abbreviations used

**Actb**: Beta actin

**BLAST**: Basic Local Alignment Search Tool

**bp**: Base pair

**BRG**: University of Louisville Bioinformatics Research Group

**cDNA**: Complementary DNA

**DNA**: deoxyribonucleic acid

**GAPDH**: Glyceraldehyde-3-phosphate dehydrogenase

**GB**: One billion bytes

**Hprt**: Hypoxanthine phosphoribosyltransferase

**Hspca**: Heat shock protein 1, alpha

**Htf9c**: HpaII tiny fragments locus 9C

**KB**: One thousand nucleotide bases

**NCBI**: National Center for Biotechnology Information

**oligo**: oligonucleotide

**PCR**: Polymerase chain reaction

**Ppicap**: Peptidylprolyl isomerase C-associated protein

**RT-PCR**: Reverse transcription polymerase chain reaction

**Sdf4**: Stromal cell derived factor 4

**Tuba1**: Tubulin, alpha 1

**Tubd1**: Tubulin, delta 1

**Ubb**: Ubiquitin B

**Ywhaz**: Tyrosine 3-monooxygenase/tryptophan 5-monooxygenase activation protein, zeta polypeptide

## Authors' contributions

ER coded the solution, and was involved in determining how to design primer pairs and oligonucleotides. In addition, ER was responsible for drafting the manuscript. AK participated in the design of the primer pair and oligonucleotide construction. In addition, he carried out the PCR amplifications and the DNA sequencing. AK was involved in testing and validation of MPrime. NC participated in the design of the primer pair in oligonucleotide construction as well as testing and validation of MPrime.
